# No room to roam: King Cobras reduce movement in agriculture

**DOI:** 10.1186/s40462-020-00219-5

**Published:** 2020-08-03

**Authors:** Benjamin Michael Marshall, Matt Crane, Inês Silva, Colin Thomas Strine, Max Dolton Jones, Cameron Wesley Hodges, Pongthep Suwanwaree, Taksin Artchawakom, Surachit Waengsothorn, Matt Goode

**Affiliations:** 1 Suranaree University of Technology, Nakhon Ratchasima, Thailand; 2 King Mongkut’s University of Technology Thonburi, Bangkok, Thailand; 3 Population and Community Development Association, Bangkok, Thailand; 4 Sakaerat Environmental Research Station, Nakhon Ratchasima, Thailand; 5 School of Natural Resources and Environment, University of Arizona, Tucson, AZ USA

**Keywords:** Snake, Reptile, *Ophiophagus hannah*, Elapid, Space-use, Step-selection, dBBMM, Site fidelity, Tropical

## Abstract

**Background:**

Studying animal movement provides insights into how animals react to land-use changes. As agriculture expands, we can use animal movement to examine how animals change their behaviour in response. Recent reviews show a tendency for mammalian species to reduce movements in response to increased human landscape modification, but reptile movements have not been as extensively studied.

**Methods:**

We examined movements of a large reptilian predator, the King Cobra (*Ophiophagus hannah*), in Northeast Thailand. We used a consistent regime of radio telemetry tracking to document movements across protected forest and adjacent agricultural areas. Using dynamic Brownian Bridge Movement Model derived motion variance, Integrated Step-Selection Functions, and metrics of site reuse, we examined how King Cobra movements changed in agricultural areas.

**Results:**

Motion variance values indicated that King Cobra movements increased in forested areas and tended to decrease in agricultural areas. Our Integrated Step-Selection Functions revealed that when moving in agricultural areas King Cobras restricted their movements to remain within vegetated semi-natural areas, often located along the banks of irrigation canals. Site reuse metrics of residency time and number of revisits appeared unaffected by distance to landscape features (forests, semi-natural areas, settlements, water bodies, and roads). Neither motion variance nor reuse metrics were consistently affected by the presence of threatening landscape features (e.g. roads, human settlements), suggesting that King Cobras will remain in close proximity to threats, provided habitat patches are available.

**Conclusions:**

Although King Cobras displayed individual heterogeneity in their response to agricultural landscapes, the overall trend suggested reduced movements when faced with fragmented habitat patches embedded in an otherwise inhospitable land-use matrix. Movement reductions are consistent with findings for mammals and forest specialist species.

## Introduction

Examining animal movement can provide important information on conspecific interactions [1], predator-prey dynamics [2, 3], reproductive behaviours [4], and responses to anthropogenic threats [5, 6]. Additionally, and perhaps most important to conservation planning, is the connection between movement and resource acquisition [7–9]. Understanding habitat requirements, via animal movement, can help prioritise the areas to protect from land-use conversion, inform management, and build conservation plans [10].

Anthropogenic land-use can alter the landscape’s ecology, changing resources [11], modifying behaviour [12], and introducing novel threats [13]. Such changes can result in increased mortality of species, or subtler sub-lethal costs [14–16]. A global review of non-volant mammals revealed that movements are impacted by human-dominated landscapes, resulting in altered movement patterns [17].

Despite indications of overall reductions in vagility, the impacts of anthropogenic landscapes on threatened species are likely to vary. Evolutionary history and key life history traits are likely to modify a species’ movements in relation to human-dominated landscapes [18]. For example, species that evolved in continuous habitat (e.g., forest specialists) likely experienced lower costs to large movements and crossing habitat barriers, potentially resulting in species leaving prime habitat and using riskier anthropogenic landscapes [18].

Vulnerability in anthropogenic landscapes can be augmented by traits such as large body size, parental investment in offspring, habitat specialisation, and low population densities, all of which have been connected to increased extinction risk [19–23]. Species frequently involved in human-wildlife conflict are also more vulnerable to direct mortality in anthropogenic landscapes [24–26].

We aimed to explore the movement patterns of a large-bodied, highly persecuted predator in a mixed-use landscape. The role of reptiles in ecosystems is underappreciated [27] and likely constitute an important aspect of the remaining wildlife in Southeast Asian agricultural systems. Few reptile species fulfil similar ecological functions comparable to large mammals, but King Cobras (*Ophiophagus hannah* [CANTOR, 1836]) share several traits with large mammals that suggest their importance in ecosystem functioning and vulnerability to habitat modification. Using radio telemetry, we assess how King Cobra modify their movements in agricultural areas via three approaches: 1) Changes in movement magnitude using dynamic Brownian Bridge Movement Model motion variance; 2) Non-random movement in relation to landscape features using Integrated Step-Selection Functions; 3) Changes in site fidelity (residency time and revisit count) using Bayesian regression models.

## Methods and materials

### Field methods

We studied King Cobras at the Sakaerat Biosphere Reserve located in Nakhon Ratchasima province, Northeast Thailand (14.44–14.55°N, 101.88–101.95°E). The reserve comprises three zones varying in levels of human-modification: the core zone, protected and fully forested; the buffer zone, protected and undergoing reforestation; and the transitional zone, an agricultural matrix dominated by rice, corn and sugar cane. The transitional zone also contains 159 villages and a four-lane highway that connects Nakhon Ratchasima to Bangkok. Further descriptions of the study site can be found in Silva et al. [28] and Marshall et al. [25, 29, 30].

Due to King Cobras’ low detectability, we used a myriad of methods: unstandardised surveys, trapping, opportunistic encounters, and village notations. Upon capture, basic morphometrics were collected (snout-vent length, mass, and sex via cloacal probing [31];) and radio transmitters were implanted (AI-2 T or SI-2 T, Holohil Inc., Ontario, Canada; following Reinert and Cundall [32]) while the snake was anesthetized using vaporized isoflurane. Further details, and morphometric measurements can be found in Marshall et al. [25, 29]. We named every individual according to their age class, sex and capture number (e.g. AM006 = an adult male who was the sixth King Cobra captured). We tracked individuals four times a day, with approximately 4 h between tracks from 2014-03-22 to 2018-07-28 (06:30, 11:00, 16:00, 20:00; the distribution of time lags between tracking is available in Supplementary Figure 1). We determined snake location using triangulation, while attempting to maintain a minimum distance of 10 m. At each location we recorded time, location (Universal Transverse Mercator 47 N WGS 84 datum), and GPS error. We obtained GPS error from handheld GPS units (Garmin 64S) after we determined the triangulated position. An alternative description of our tracking protocols can be found in Silva et al. [28].

### Environmental data

We obtained daily rainfall and temperatures from five weather stations within the Sakaerat Biosphere Reserve core zone to identify seasons [33]. We averaged daily readings by station, and ran cluster analysis to generate seasons using the *segclust2D* package v.0.2.0 [34]. *segclust2D* analysis suggested that five clusters and 23 segments was the optimal way of dividing the 2012–2018 period into seasons. However, it resulted in seasons unique to single years. Therefore, we manually simplified the seasons into three groups that appeared in nearly all years: hot (x̄ = 33.8 ± 2.8 °C, x̄ = 2.5 ± 7.9 mm rainfall), wet (x̄ = 29.9 ± 2.2 °C, x̄ = 5.9 ± 11.1 mm rainfall) and dry (x̄ = 29.0 ± 3.5 °C, x̄ = 0.2 ± 0.8 mm rainfall). Standard errors (SE) associated with x̄ are indicated by ± (calculated using the *pracma* package v.2.2.5 [35]).

We obtained land-use shapefiles from a land survey by the Thai Land Development Department [36] that covered the entire study site. We converted categorical land-use classifications to continuous raster layers, describing Euclidean distances to key landscape features (i.e., forest, roads, semi-natural areas, settlements, and water bodies). We set the cell size of the newly created rasters to approximately 10 m, which was sufficiently small to detect fine-scale changes. We selected forests, because they constitute an important habitat of King Cobras and are the least disturbed habitat type in the study area. We selected settlements and roads because they pose direct threats to King Cobras via human-snake conflict or vehicle collisions. We selected water bodies and semi-natural areas because they contained natural vegetation, potentially providing cover and/or prey items for King Cobras within the agricultural matrix. We classed semi-natural areas as areas of scrub and vegetation not actively being farmed, often along field margins, irrigation canals and in disused plots.

### Motion variance and area estimation

Although criticised, research on terrestrial reptile spatial ecology has relied on kernel density and minimum convex polygon approaches to estimate space-use, as a proxy for movements and activity [37, 38]. Kernel density estimators are problematic, because the technique assumes independence between locations, which can never be strictly met in radio tracking datasets [39]. Efforts to combat autocorrelation [40], lead to a loss of information decreasing the biological relevance of space-use estimates [41].

Dynamic Brownian Bridge Movement Models (dBBMMs, [42]) present an alternative that accounts for non-independence of locations and provides a balance between over- and under-estimating space-use [28, 38]. We ran Dynamic Brownian Bridge Movement Models to estimate motion variance and the space-use of King Cobras (*move* package v.3.1.0 [43]). From the dBBMM occurrence distributions we extracted several contours to represent space-use (using R packages *adehabitatHR* v.0.4.16 [44], and *rgeos* v.0.4.2 [45]). The selection of a suitable space-use contour can be considered arbitrary, so we opted to report a range of contours (90, 95, and 99%) that help to describe the overall space-use during the study period, while also displaying the sensitivity to contour selection. We used dBBMMs instead of standard BBMMs, because the former allowed for estimates of changes in motion variance over time [42, 46]; therefore, providing a proxy for reptile movement that we could compare between land-use types. Following Kranstauber et al. [42], we selected a window size of 25 and margin size of 5 for dBBMMs based on a timeframe that was biologically relevant to suspected changes in behavioural states. Due to our reliance on radio tracking and associated coarse temporal resolution data, we targeted the identification of activity and sheltering. We were able to detect shifts from activity to sheltering with slightly greater than 1 day of radio tracking effort; therefore, we set margin size at 5 data points. A relevant time for a behavioural state to last was approximately 1 week (i.e., long-term sheltering); therefore, we set window size to 25 data points. We used the GPS error, taken from hand held units used in the field, for dBBMM location error on a point-by-point basis; for points that did not have GPS error recorded we used the mean GPS error for that individual.

We explored seasonal changes in motion variance and how it was impacted by an individual’s proximity to landscape features (i.e., forest, roads, semi-natural areas, settlements, and water bodies). Due to serial autocorrelation and over dispersal in motion variance and distance raster values, we used non-metric multidimensional scaling (NMDS) to explore interactions among these variables because NMDS is subject to fewer assumptions regarding data structure and relationships between variables. We ran NMDS on a distance matrix created from rasters that described distances from key landscape features (using 2000 iterations to produce two axes; using the *vegan* package v.2.5.5 [47]). We plotted the resulting two-dimensions and coloured points corresponding to the motion variance values. The resulting visualisation allowed us to identify areas of high or low motion variance and the manner in which they are associated with the distance of snakes to landscape features.

### Integrated step-selection function

To explore King Cobra avoidance of or attraction to key landscape features, we used Integrated Step-Selection Functions (ISSF) from the *amt* package v.0.0.6 [48]. Integrated Step-Selection Functions are a method of assessing habitat preference in animals, comparing used locations to those randomly placed within the landscape. By using movement parameters to help guide the random locations, ISSF have been shown to produce more stable estimates of animal preference under different model structures and landscape configurations, while allowing estimation of movement-habitat interactive effects [49].

We used the same distance from landscape features rasters as in the above NMDS analyses. For ISSF, we inverted raster layers to avoid zero-inflation in distance values and make interpretation of resulting effects more intuitive (i.e., negative effect = avoidance). We used the landscape values at the endpoints in ISSF, because our sampling regime was temporally insufficient to assume straight-line movements between locations (approx. 4 times a day). We produced 200 random locations per step, with no resampling of data, because temporal resolution of our radio tracking data was coarser than GPS data allowing high numbers of random steps without requiring prohibitively intense computation. Producing 200 random locations reduced the chance of missing rare landscape types, and made the best use of the high-resolution raster data [50].

All nine models included step length and angle [51], with random step lengths and angles drawn from gamma (only positive step lengths) and von Mises (angles) distributions, respectively. One model only included step length and angle as predictors, five models included step length, angle and distance from a landscape feature, and three models included step length, angle and a combination of distances from multiple uncorrelated landscape features interacting with step length and angle. We selected models per individual using Aikike’s Information Criterion (AIC), considering those with ∆ AIC < 2 as top performing [52]. We did not model average to produce a population level model, because we observed high individual heterogeneity. We excluded AM007 from the ISSF analysis, because he never left forested areas.

### Site fidelity and reuse

Shelter sites are important for species requiring extended periods of low mobility to digest meals [53] or undergo ecdysis [54]. Using an intensive radio tracking regime allowed us to identify individual shelter sites, time spent within shelters, and frequency of reuse. We examined how site-reuse patterns changed in relation to anthropogenic landscape features.

We identified site reuse, expressed as the number of revisits, residency, entrance and exit times of visits, and a unique ID for each site, with the *recurse* package v.1.1.0 [55]. We defined each site as a circular area with a radius equal to the mean GPS error recorded for each individual (x̄ = 5.1 ± 0.8 m, range = 3.5–10.0 m) centred on each unique location. When examining the frequency of revisits, we filtered out sites where the snake was present for less than the mean time between radio telemetry data points (9 h).

Time spent at sites (residency time) and the reuse rate (revisit count) have direct connections to the extent of animal space use, making them useful metrics to detect movement restrictions [56]. To explore changes in our two chosen site fidelity metrics (residency time and revisit count), we ran four Bayesian regression models in JAGS using the *jagsUI* package v.1.5.0 [57]. Two models used a log normal distribution to explore the impacts of proximity to uncorrelated landscape features on log transformed residency time [55]. Two models used a Poisson distribution to explore the impacts of proximity to uncorrelated landscape features on revisit counts [55]. We determined spatial correlation in the landscape rasters and created two groups of uncorrelated variables (*r* < 0.6) to use as predictors: 1) roads, forest, and settlements; 2) roads, forest, and semi-natural areas.

We used individual ID as a random effect impacting both models’ intercepts and gradients to avoid pseudoreplication. We excluded AM007 from models because he remained in the forest; therefore, had little opportunity to display preference beyond forests or interact with landscape features.

We used Cauchy (location = 1, scale = 1) and half Cauchy distributions [58] as hyperparameters for the centre and precision of normal distributions priors for individual random effects on distance to forest, semi-natural areas, roads, settlements and water bodies. We selected weakly informative priors based on the assumption that King Cobras would follow similar movement patterns as those described in Tucker et al. [17]: which were characterised by reduced movement associated with anthropogenic features. We assigned a negative value to the prior for the effect of distance to forest negative, reflecting the likely opposite effect from proximity to anthropogenic features. We ran all models using three chains over 20,000 interactions, with the first 5000 discarded as burn-in and a thinned factor of 50. Full JAGS models specifications can be found at DOI: 10.5281/zenodo.3905757.

We identified convergence via $\hat{R}$ values and traceplots. We evaluated model performance using Bayes *p*-values, followed by visual inspection of posterior predictive check plots.

The *recurse* package also allowed us to quantify time spent in the protected core zone of the reserve. Comparing movements to a shapefile of the reserve’s protected zone allowed us to create a summary of all boundary crossings (entrance and exit times). From the revisit data, we calculated overall time spent in the core zone and plotted the use of the zone over time, allowing us to illustrate how King Cobras interact with barriers.

### Software and data

We completed all analysis in R v.3.5.3 [59] and R Studio v.1.2.1335 [60]. The full dataset, with code workflow scripts, can be found at DOI: 10.5281/zenodo.3905757. Movement data is also available on MoveBank (Movebank ID: 1093796277).

For data manipulation, we used R packages *broom* v.0.5.2 [61], *data*.*table* v.1.12.2 [62], *dplyr* v.0.8.3 [63], *forcats* v.0.4.0 [64], *lubridate* v.1.7.4 [65], *openxlsx* v.4.1.0 [66], *readr* v.1.3.1 [67], *reshape2* v.1.4.3 [68], and *stringr* v.1.4.0 [69]*.* We handled rasters and shapefiles with R packages *raster* v.2.8.19 [70], *rgdal* v.1.4.3 [71] and *sp* v.1.3.1 [72, 73]. For visualisations we used R packages *cowplot* v.0.9.4 [74], *ggplot2* v.3.2.1 [75], *ggpubr* v.0.2 [76], *ggspatial* v.1.0.3 [77], *scales* v.1.1.0 [78] and *scico* v.1.1.0 [79]. To determine model convergence and evaluate model performance, we used the R packages *ggmcmc* v.1.2 [80], *ggridges* v.0.5.1 [81], and *tidybayes* v.1.0.4 [82].

## Results

**Fig. 1:**
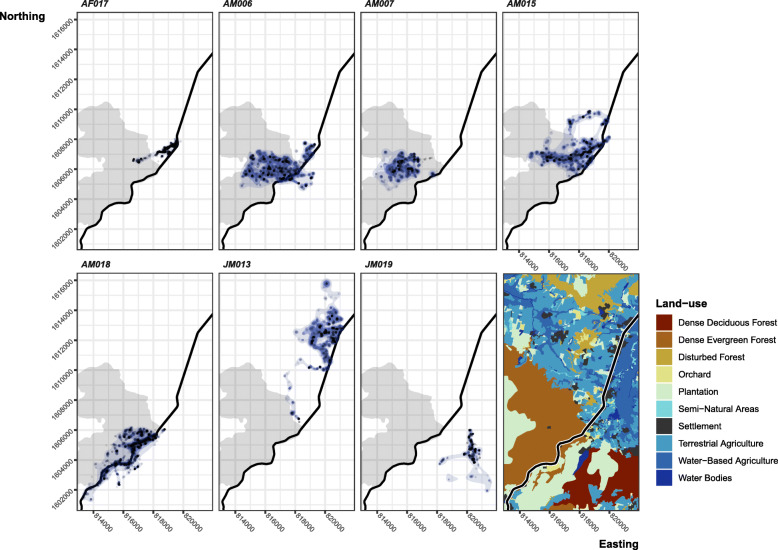
Dynamic Brownian Bridge Movement Model estimates of utilisation distribution contours. Areas displayed with increasing levels of opacity are the 99, 95 and 90% utilisation contours. Black dots show locations. The shaded background area shows the protected core area. Dark central line is the four-lane 304 highway. Bottom right map shows the land-use types in the area

We tracked seven King Cobras for an average of 649.7 ± 112.3 days (Table 1). We tracked and located each King Cobra an average of 1834 ± 297.1 times, with an average of 8.5 ± 0.1 h between fixes (range = 0.1–793.9 h; Supplementary Figure 1). King Cobras occupied an average of 524 ± 104.5 unique locations, covering large areas in both protected and unprotected areas (Table 1; Fig. 1), with adult males tending to move more than our single tracked female and two tracked juveniles. The two juvenile males differed greatly from each other, likely the result of JM013’s northward travel.

**Table 1:** Summary of tracking and movements

ID	Datapoints	Days	Unique locations	Revisit frequency	Time stationary	dBBMM Range (ha)			σ^**2**^m	% outside PA
						90	95	99		
**AF017**	2245	774.97	728	3.19	1.84 ± 0.13	41.69	68.15	149.28	7.53 ± 0.33	91.53
**AM006**	2173	723.05	542	19.03	2.58 ± 0.27	519.60	701.44	1063.42	42.61 ± 1.74	15.49
**AM007**	969	320.66	220	12.83	2.61 ± 0.53	232.70	345.62	616.90	51.90 ± 3.81	0.67
**AM015**	1944	680.13	587	13.60	2.24 ± 0.22	379.80	603.32	1081.54	27.3 ± 1.22	67.57
**AM018**	3122	1176.10	985	7.79	2.16 ± 0.14	255.09	492.54	977.84	33.56 ± 1.41	42.39
**JM013**	1497	561.19	381	21.58	3.09 ± 0.39	354.33	533.26	972.74	22.35 ± 1.11	99.95
**JM019**	890	311.79	228	11.99	2.56 ± 0.37	61.01	119.04	390.39	7.90 ± 0.63	100.00

*Data points* Number of data points collected on an individual irrespective of move or not, *Unique locations* Number of unique locations visited by an individual, *Days* Number of days tracked, *Revisits frequency* The number of days between revisits to a previously used location (days tracked / count of reused locations), *Time stationary* Mean sheltering time ± SE in days, *dBBMM Range* Range areas estimated using dBBMM 99, 95, and 90% contours, *σ*^*2*^*m* Mean motion variance ±SE, *% Outside of PA* Percentage of total time tracked an individual was outside of the protected area

**Fig. 2:**
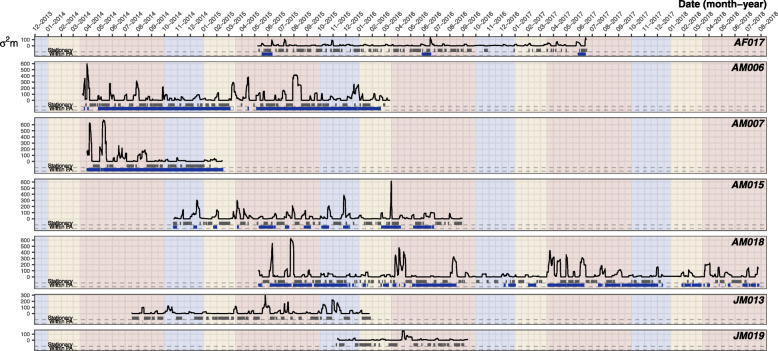
Motion variance of each individual over their tracking period. Black lines show the motion variance values over time. Grey bars indicate long-term sheltering behaviour (i.e., when the time sheltering was greater than the individual’s mean sheltering time). Blue bars indicate times when the individual was within the protected forested area. Shading shows the three seasons: red = Hot, blue = Wet, yellow = Dry

Time spent in human disturbed areas varied dramatically between individuals and showed modest seasonal patterns (x̄ = 59.7 ± 15.5%, range = 0.7–100%; Table 1, Fig. 2). During the start of the hot season (Fig. 2 red highlight, February–April), adult males ventured out of protected forested areas, a pattern particularly clear in AM006’s movements. During other times of the year, snakes exhibited more consistent use of the protected area, which coincided with more frequent long-term use of shelter sites (Fig. 2). The female, AF017, showed a consistent yearly pattern of entering the protected area via a semi-natural area corridor that connected to a streambed.

### Motion variance

Mean motion variance differed among individuals (x̄ = 27.9 ± 0.6 m, range = 5.6e^− 05^ – 675.8 m, Table 1). The largest motion variances belonged to adult males, characterized by larger movements concentrated at the beginning of the hot season (Fig. 2, red highlight). Juvenile males did not move as far as adult males at any time of the year, but they did appear to be more active than the female, AF017. Motion variance of AF017 peaked during the hot season, when she entered the protected area in mid- to late-April and left in mid-May. Overall individuals displayed seasonal differences in motion variance, with lower values during the dry season (x̄ = 14.0 ± 0.6 σ^2^m) compared to hot (x̄ = 34.9 ± 1.0 σ^2^m) and wet seasons (x̄ = 22.1 ± 0.8 σ^2^m; Fig. 2).

Motion variance was highest in evergreen and disturbed forests (x̄ = 38.9 ± 1.1, max = 665 m; x̄ = 48.3 ± 4.8, max = 598 m), and lowest in orchards (x̄ = 10.5 ± 1.24 m, max = 449), semi-natural areas (x̄ = 11.6 ± 0.6, max = 347 m), and water bodies (x̄ = 10.3 ± 1.4, max = 119 m; Supplementary Figure 2).

**Fig. 3:**
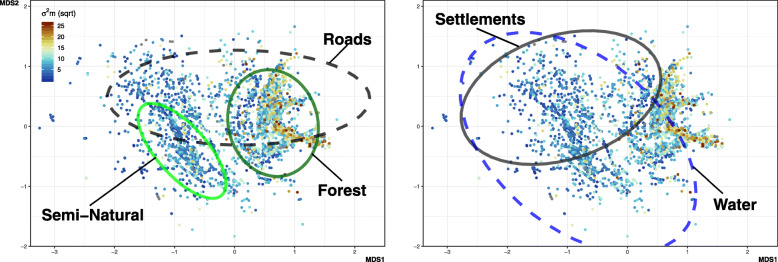
Non-metric multidimensional scaling plot. Motion variance values are reflected by the colour of the points, we have rooted these values so value differences are easier to distinguish. Ellipses indicate 95% of points within 100 m of a given landscape feature. **a** Ellipses highlight points existing within 100 m of forest, semi-natural areas, and roads. **b** Ellipses highlights points existing within 100 m of water, and settlements

Using NMDS, we successfully reduced the dimensionality of distance to chosen landscape features, revealing several patterns. Higher motion variance values were mostly associated with when snakes were < 100 m from forested areas (Fig. 3; see Supplementary Figure 3 for bi-plot). This was the clearest pattern. In contrast to motion variance values near or within forests, NMDS revealed lower values when close to semi-natural areas (< 100 m). All other covariates were less associated with particular motion variance values. Roads contained a wide array of values, which overlapped with forest and semi-natural areas, suggesting weaker impacts on motion variance. Settlements and water bodies revealed similarly weak associations to motion variance, but there was a tendency for motion variance near or within settlements to be lower than those near or within forests.

### Integrated step-selection function

**Fig. 4:**
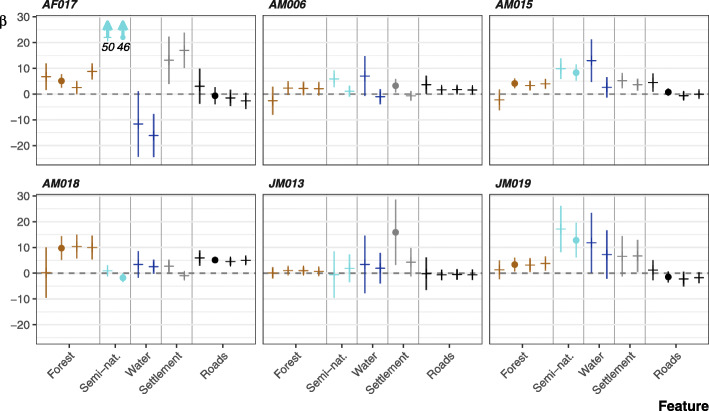
The coefficients from the integrated step-selection functions per individual. Error bars show the 95% confidence interval. Circles indicated effects included in the top model as determined by ∆ AIC. Positive effects indicate a positive association towards a landscape feature as the distance values were inverted

**Fig. 5:**
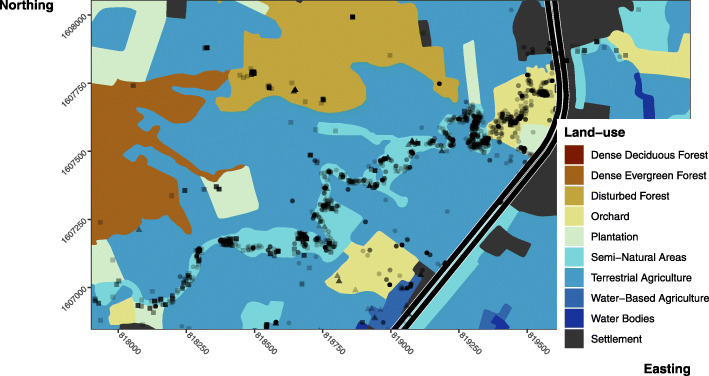
A map of land-use illustrating how King Cobra movements are largely occurring within semi-natural areas. Displayed using semi-transparent points, are the locations of King Cobras across the entire study period. Circles = AF017, triangles = AM006, squares = AM015

Individual movements of the King Cobras were best described by three models (Fig. 4; Table 2; full ISSF results can be found in Supplementary Table 2). Model 7 performed best for four individuals, and included proximity to forest, roads, and semi-natural areas. The locations of AF017, AM015, AM018 and JM019 were positively associated with forests. However, the association between movements, roads, and semi-natural areas varied; AF017, AM015 and JM019 prefered semi-natural areas, but were inconsistently associated with roads. The movements of AM006, AM015, and AF017 while in agricultural land exemplifies King Cobras’ reliance on semi-natural areas (Fig. 5). By contrast, AM018’s locations were associated with roads, while weakly avoiding semi-natural areas. But for AM018 model 8 was within 2 ∆ AIC. Model 8 replaced semi-natural areas with settlements as a predictor, indicating positive association (ß = 2.504, 95% CI -0.244 – 5.253). Models targeting JM013’s movements were similarly inconclusive, with four models achieving ∆ AIC < 2 (including the null step and angle only model), indicating distance to landscape feature was a poor predictor of movement. Finally, AM006’s movements were best described by model 6, indicating a weak association with water bodies.

**Table 2:** ISSF model formulation and AIC results

Model	Model formula, all begin with Model1 formulation	AF017	AM006	AM015	AM018	JM013	JM019
Model1	log_sl*cos_ta + strata (step_id_)	7573.47	5794.50	6205.87	10,567.77	**4014.21**^**a**^	2402.41
Model2	Model1 + dist_forest+dist_forest:log_sl + dist_forest:cos_ta	7555.47	5794.62	6187.03	10,530.19	**4013.58**^**a**^	2399.25
Model3	Model1 + dist_settle+dist_settle:log_sl + dist_settle:cos_ta	7542.05	5794.64	6201.81	10,552.30	**4014.60**^**a**^	2402.45
Model4	Model1 + dist_semiNat+dist_semiNat:log_sl + dist_semiNat:cos_ta	7492.52	5783.74	6186.73	10,563.71	4017.76	2392.32
Model5	Model1 + dist_road+dist_road:log_sl + dist_road:cos_ta	7557.50	5791.24	6201.98	10,498.78	4017.31	2406.31
Model6	Model1 + dist_water+dist_water:log_sl + dist_water:cos_ta	7566.34	**5779.88**^**a**^	6199.01	10,559.55	**4013.23**^**a**^	2404.11
Model7	Model1 + dist_road+dist_forest+dist_semiNat	**7471.48**^**a**^	5789.37	**6171.16**^**a**^	**10,471.26**^**a**^	4017.55	**2388.28**^**a**^
Model8	Model1 + dist_road+dist_forest+dist_settle	7539.43	5789.9	6195.03	**10,472.37**^**a**^	4017.61	2400.04
Model9	Model1 + dist_road+dist_forest+dist_water	7530.33	5789.75	6187.41	10,474.02	4015.71	2398.12

*sl* Step length, *ta* Turning angle, *dist*_* Distance from forest, settlement, semi-natural area, road, and water

^a^ Bold indicate the models < 2 ∆ AIC from the top-performing model

Looking at all models regardless of AIC, 22 of 24 models included positive associations with forest (Fig. 4). Semi-natural areas, settlements and water bodies were similarly preferred with 10 of 12, 9 of 12 and 10 of 12 models, respectively, showing positive associations across all individuals. Roads produced mixed results (12 of 24 showing positive association), with both positive and negative associations within and between individuals.

**Fig. 6:**
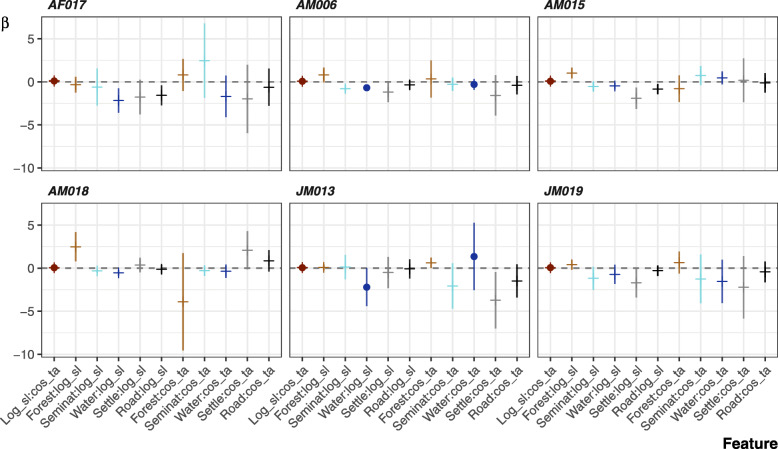
The interactive coefficients from the integrated step-selection functions per individual. Error bars show the 95% confidence interval. Circles indicated effects included in the top model as determined by ∆ AIC. log_sl = step length, cos_ta = turning angle. Positive effects indicate a positive interaction between step or angle with a landscape feature as the distance values were inverted

The top performing models for four modelled individuals (AF017, AM015, AM018, JM019) did not include any interactive effects. The two that contained interactive effects (AM006, JM013), revealed that as these individuals neared water bodies, they tended to exhibit shorter step lengths (Fig. 6). Outside of the top performing models, we observed negative relationships between step length and semi-natural areas (5 of 6 individuals), water bodies (6 of 6), settlements (5 of 6), and roads (6 of 6), whereas forests were related to increased step lengths in five individuals. Interactions with turning angle are more uncertain, but broadly show increased turning angles when nearer to semi-natural areas (4 of 6 individuals), water bodies (4 of 6), settlements (4 of 6), and roads (5 of 6); with lower turning angles when nearer forests (4 of 6).

### Site fidelity and reuse

The *recurse* analysis revealed that mean time spent at a shelter site (stationary for more than 9 h) was 2.3 ± 0.1 days (range = 0.4–43.5 days; Supplementary Figure 4), and all snakes demonstrated site fidelity, revisiting a previously used site on average every 13.0 ± 2.4 days (range = 3.2–21.6 days; Table 1).

All models we ran to predict residency time and revisits converged and produced Bayes *p*-values from 0.52 to 0.58.

Data revealed weak negative relationships between distance to roads and residency time for AM015 and JM019, indicating longer stationary periods when nearer to roads, but 95% Credible Intervals overlapped zero (Supplementary Figure 5). Distance to forests appeared not to impact residency time, all individual estimates overlapped zero, with small 95% Credible Intervals (between − 0.183 and 0.142). There were weak negative relationships between residency time and distance to semi-natural areas in four individuals (AM015, AM018, JM013, JM019). However, only AM018’s 66% Credible Interval (approximately 1 standard deviation) excluded zero. Evidence for AF017 and AM006 reducing residency time when near semi-natural areas was weak, only AM006 66% Credible Intervals excluded zero.

Compared to residency time, revisits model estimates were more centred on zero, with all 66% Credible Intervals of ß estimates including zero (Supplementary Figure 5). The ranges of Credible Intervals associated with revisitation were smaller than those estimating impacts on residency time (between − 0.059 and 0.060), indicating greater confidence in the non-effect of landscape features on site revisits. Complete results of all Bayesian models can be found in Supplementary Table 1.

## Discussion

We present evidence on how a large tropical reptile modifies its movements when in agricultural areas. We documented: 1) reductions in movement magnitude, 2) significant non-random movement, 3) and no changes in site fidelity (residency time and revisit count), in relation to landscape features (forest, roads, semi-natural areas, settlements, and water bodies).

Motion variance was characterized by seasonal peaks associated with breeding activity, but generally showed decreased activity in agricultural areas. Reduced movement in anthropogenic systems is consistent with broad findings on mammalian movements in anthropogenic systems [17]. Research on reptile spatial ecology has documented either reduced space-use [83–86] or reduced movement [9, 87–89] in fragmented agricultural landscapes, but the response to fragmentation is not universal. Related research failed to detect significant shifts in movement patterns [90–92], or revealed increased space-use in fragmented areas [93, 94], potentially due to species’ life history traits [18]. King Cobras appear to react in a manner consistent with forest specialists, or taxa that have evolved in continuous habitat [18], characterised by limited boundary avoidance, large movements, and mortality in human-dominated areas [25]. Habitat-specialist species occupying fragmented areas likely face limited resources, resulting in restricted movements to more naturalistic corridors [95].

The clearest pattern we documented was preferential use of semi-natural vegetation patches, compared to the surrounding matrix of agricultural fields, when King Cobras moved within agricultural areas. These patches primarily consist of dense vegetation arrayed linearly along the banks of irrigation canals, acting as movement corridors through the fragmented landscape. Linear habitats potentially impact movements in other reptile species [9, 96]. Doherty et al. [9] suggested that reduced movement by Eastern Bearded Dragons (*Pogona barbata*) was partly driven by higher prey availability in linear vegetation patches. Although we lack direct evidence suggesting semi-natural areas within agricultural landscapes host relatively higher prey abundance, it is likely King Cobra prey can be found more frequently where vegetation and water are present [30, 97, 98]. However, increased movements in forests, at least for some individuals, may indicate that resource availability alone fails to explain variation in movement patterns. Intact forests are extremely valuable and present a resource-rich environment, theoretically reducing the need for foraging movements [99, 100].

Ectotherms also have to consider the thermal qualities of habitats, shifting habitat usage to maximise thermoregulatory efficiency and achieve optimal temperatures [101, 102]. Compared to temperate regions, evidence from the tropics that behavioural shifts are required to maximise thermoregulation is more ambiguous [103], but not unknown in larger species [104]. Open fields and vegetation corridors present two contrasting thermal environments. When temperatures are high, tropical snakes may need to seek out cooler, shaded micro sites in areas that are richer in shelter sites.

Utilisation of covered areas may also be tied to threat avoidance, as threats are known to influence animal movement [105, 106]. In our study area, roads pose a major threat to many animals [107], and King Cobras routinely exit the protected area. Outside of the protected area, King Cobras fall victim to both direct [25] and indirect [108] human-caused mortality. However, we failed to detect clear avoidance of roads or human settlements despite reduced movement. King Cobras made use of habitat patches regardless of their proximity to threatening landscape features. Similarly, patterns of site reuse remained consistent with respect to proximity to landscape features, suggesting that the overarching driver of site residency time and revisitation is largely independent of habitat, and more likely connected to cycles of ecdysis, prey capture and digestion [53, 54].

Despite support for patterns of reduced movement by King Cobras in agricultural landscapes, we encourage caution when extrapolating these findings beyond the study sample. Our sample is likely affected by biases in Trappability & self-selection, Acclimation & habituation, and Genetic make-up (following the categories defined by Webster & Rutz [109]).

Trappability & self-selection: Our small sample for motion variance analysis (*n* = 7) and step-selection and site reuse analyses (*n* = 6), likely skewed towards individuals willing to enter settlements, was a result of low capture rates and likely low density of King Cobras. Even with a small sample and considerable tracking lengths, we observed considerable individual variation that confounds population level generalisations. Given the behavioural flexibility we observed, the diversity of habitats King Cobras inhabit across Southeast Asia (rural, forest, urban), and differences in study design [29, 110], we caution against direct comparisons between our findings and King Cobra studies in other areas.

Acclimation & habituation: While we tried to minimise the effects of frequent tracking on our tracked King Cobras by attempting to stay at least 10 m away when determining locations; repeated contact with humans over a prolonged period of time may have lessened the studied King Cobra’s aversion to humans, dampening their reaction to anthropogenic landscape features. Alternatively, during radio telemetry tracking King Cobras may be driven to shelter more frequently to avoid human contact.

Genetic make-up: The study site represents a reproductive population of King Cobras based on observations of tracked individuals mating. However, we have limited knowledge of how our sampled individuals are related. We cautiously suspect that the low detectability of King Cobras reflect a low population density (studies incorporating detection probability would be necessary to confirm this suspicion); if true, we expect a greater diversity of responses to anthropogenic landscapes in larger more genetically diverse populations or across the King Cobra’s distribution.

Despite the limitations discussed above, our results can inform hypotheses and provide priors (albeit weak priors given the small sample) for future examinations of reptile movement in relation to human landscapes, whether further field studies or meta-analyses. Further, changes in site reuse may be present at finer temporal scales than the intensity of our tracking regime. Long-term GPS tracking of snakes presently appears unfeasible [111]. Technological advances may enable more intensive and consistent tracking of individuals, allowing for identification of subtler behavioural and movement responses to anthropogenic landscapes.

Building on our results, we suggest that future conservation research focus on landscape connectivity. Irrigation canals and forest fragments may allow King Cobras to persist across areas largely separated from protected forest. Research on landscape connectivity could be especially beneficial if paired with an assessment of how threats can be effectively mitigated. The apparent lack of threat avoidance illustrated by King Cobras in this study demands changes in human behaviour. For example, road crossing structures in combination with fencing would likely help to mitigate the threat posed by roads [112]. Whereas reducing persecution of King Cobras will require a change in current negative perceptions [24, 25] and improvements in humane snake removal services, although the cost-effectiveness of snake removal services needs further quantitative study [113].

## Conclusion

Our results indicate that limited areas in agricultural landscapes are suitable for King Cobras in our sample, resulting in reduced movements that largely occur within vegetation patches along irrigation canals. Apparent reliance on vegetated patches, in an otherwise hostile human-dominated matrix, mirror findings that landscape heterogeneity and the presence of semi-natural vegetated features are required to maintain reptile diversity [114–116]. The vulnerability of King Cobras in agricultural areas suggests that these areas may be acting as a population sink [25, 117], which emphasises the importance of maintaining vegetated areas within the landscape matrix to provide refuge from known mortality sources, and movement corridors between larger habitat patches. More broadly, our findings suggest that wide-ranging reptiles can react to landscape fragmentation in similar ways to terrestrial mammals. This is especially important, because large snakes, such as King Cobras, fulfil underappreciated ecosystem roles [27, 118]. Their role in top-down trophic structuring may be comparable to predatory mammals.

## Supplementary information

**Additional file 1: Supplementary Figure 1.** Distribution of time lags between radio tracking fixes. Dashed lines indicate the mean time lag. X scale is log transformed and clipped at 96 h for ease of visualisation. **Supplementary Figure 2.** Motion variance values in each habitat type displayed as box and violin plots. Circles are the mean motion variance values for each habitat. Y-axis scale is log. **Supplementary Figure 3.** Bi-plot of NMDS results. Motion variance values are reflected by the colour of the points, we have rooted these values so value differences are easier to distinguish. **Supplementary Figure 4.** Distribution of sheltering periods. To help distinguish individual lines the plots has been split in two. The top plot shows the results from the adult males: AM006, AM007, AM015 and AM018. The lower plot shows AF017, JM013 and JM019. **Supplementary Figure 5.** Coefficient point estimates and 95% credible intervals from Bayesian regression models. Each point and line denote an individual’s point estimate and credible intervals for the impact of distance to landscape feature on residency time and revisit number. **Supplementary Table 1.** All co-efficient results from Bayesian logistic regression models. **Supplementary Table 2.** Full ISSF results for all models and individuals.

## Data Availability

Data used in this study is available on Zenodo (DOI: 10.5281/zenodo.3905757) and Movebank (Movebank ID: 1093796277). The Zenodo repository also includes all R scripts used to run analysis and generate figures.
