# Supplementary material for: No room to roam: King Cobras reduce movement in agriculture
Source: Mov Ecol. 2020 Aug 3;8:33. doi: 10.1186/s40462-020-00219-5 (PMC7397683; doi:10.1186/s40462-020-00219-5)
Supplement: Supplementary file 1 — Additional file 1: Supplementary Figure 1. Distribution of time lags between radio tracking fixes. Dashed lines indicate the mean time lag. X scale is log transformed and clipped at 96 h for ease of visualisation. Supplementary Figure 2. Motion variance values in each habitat type displayed as box and violin plots. Circles are the mean motion variance values for each habitat. Y-axis scale is log. Supplementary Figure 3. Bi-plot of NMDS results. Motion variance values are reflected by the colour of the points, we have rooted these values so value differences are easier to distinguish. Supplementary Figure 4. Distribution of sheltering periods. To help distinguish individual lines the plots has been split in two. The top plot shows the results from the adult males: AM006, AM007, AM015 and AM018. The lower plot shows AF017, JM013 and JM019. Supplementary Figure 5. Coefficient point estimates and 95% credible intervals from Bayesian regression models. Each point and line denote an individual’s point estimate and credible intervals for the impact of distance to landscape feature on residency time and revisit number. Supplementary Table 1. All co-efficient results from Bayesian logistic regression models. Supplementary Table 2. Full ISSF results for all models and individuals. [file 40462_2020_219_MOESM1_ESM.pdf]

Supplementary Material

No room to roam: King Cobras reduce movement in agriculture

Benjamin Michael Marshall[1]*, Matt Crane[2], Inês Silva[2], Colin Thomas Strine[1†], Max Dolton Jones[1], Cameron Wesley Hodges[1], Pongthep Suwanwaree[1], Taksin Artchawakom[3], Surachit Waengsothorn[4], Matt Goode[5]

[1] Suranaree University of Technology, Nakhon Ratchasima, Thailand
[2] King Mongkut's University of Technology Thonburi, Bangkok, Thailand
[3] Population and Community Development Association, Bangkok, Thailand
[4] Sakaerat Environmental Research Station, Nakhon Ratchasima, Thailand
[5] School of Natural Resources and Environment, University of Arizona, Tucson, AZ, USA

* benjaminmichaelmarshall@gmail.com
† strine.conservation@gmail.com

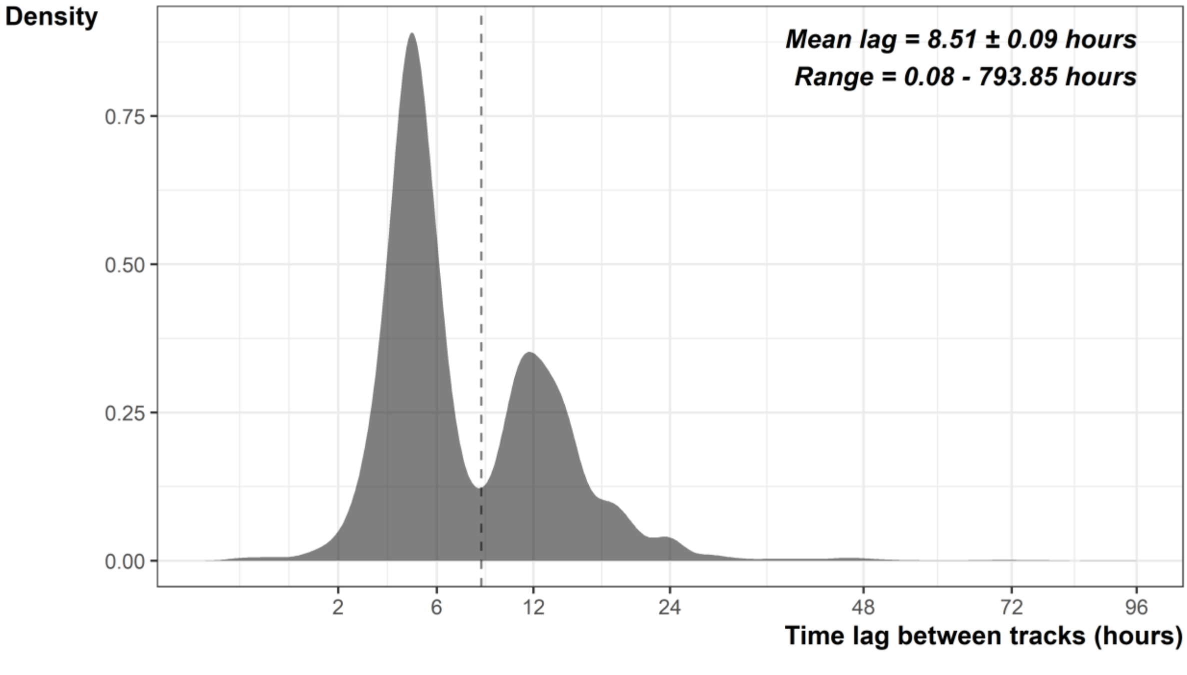

Supplementary Figure 1. Distribution of time lags between radio tracking fixes. Dashed lines indicate the mean time lag. X scale is log transformed and clipped at 96 hours for ease of visualisation.

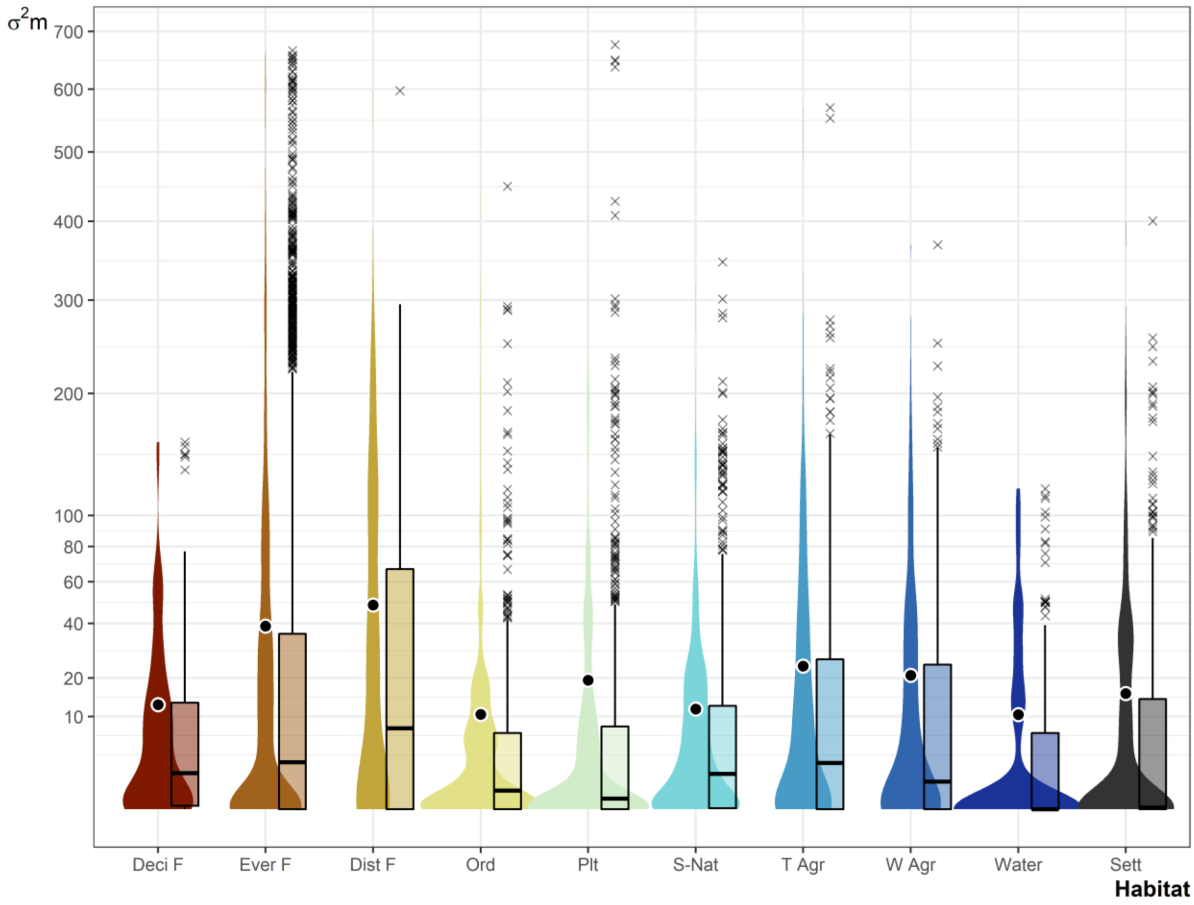

Supplementary Figure 2. Motion variance values in each habitat type displayed as box and violin plots. Circles are the mean motion variance values for each habitat. Y-axis scale is log.

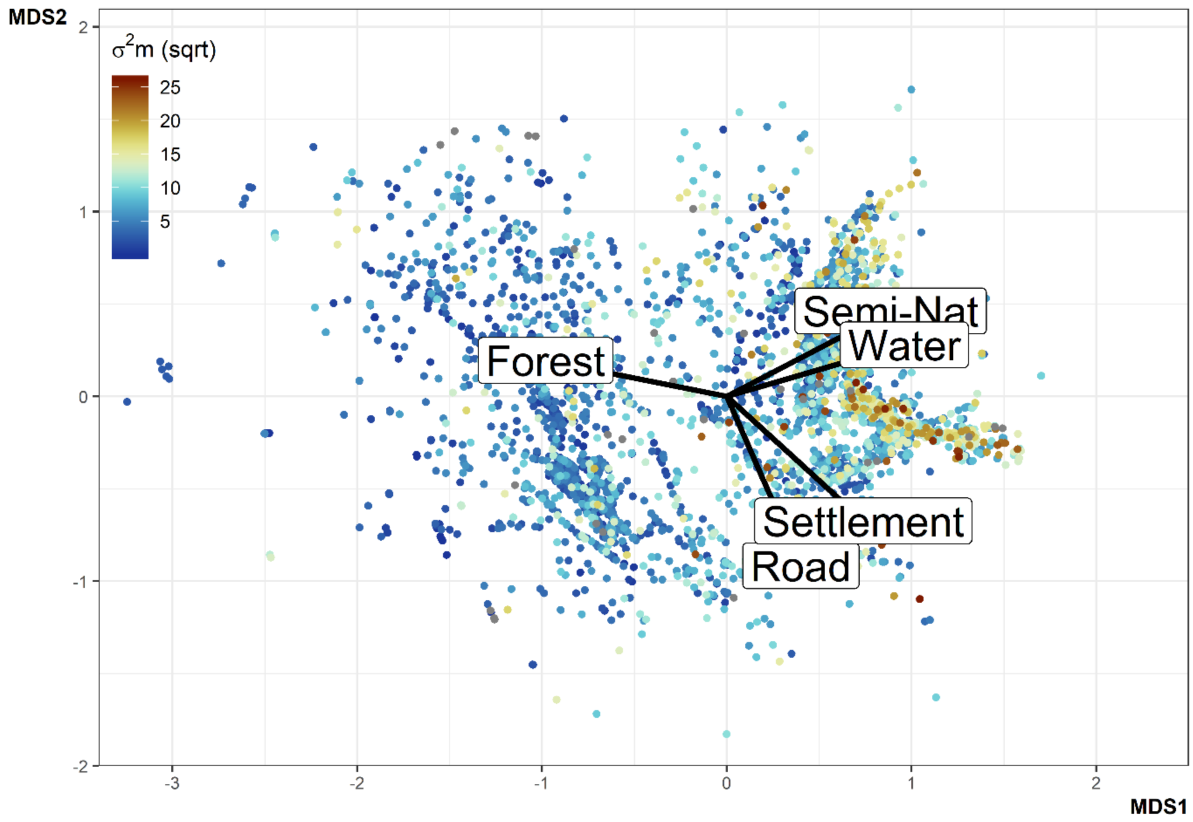

Supplementary Figure 3. Bi-plot of NMDS results. Motion variance values are reflected by the colour of the points, we have rooted these values so value differences are easier to distinguish.

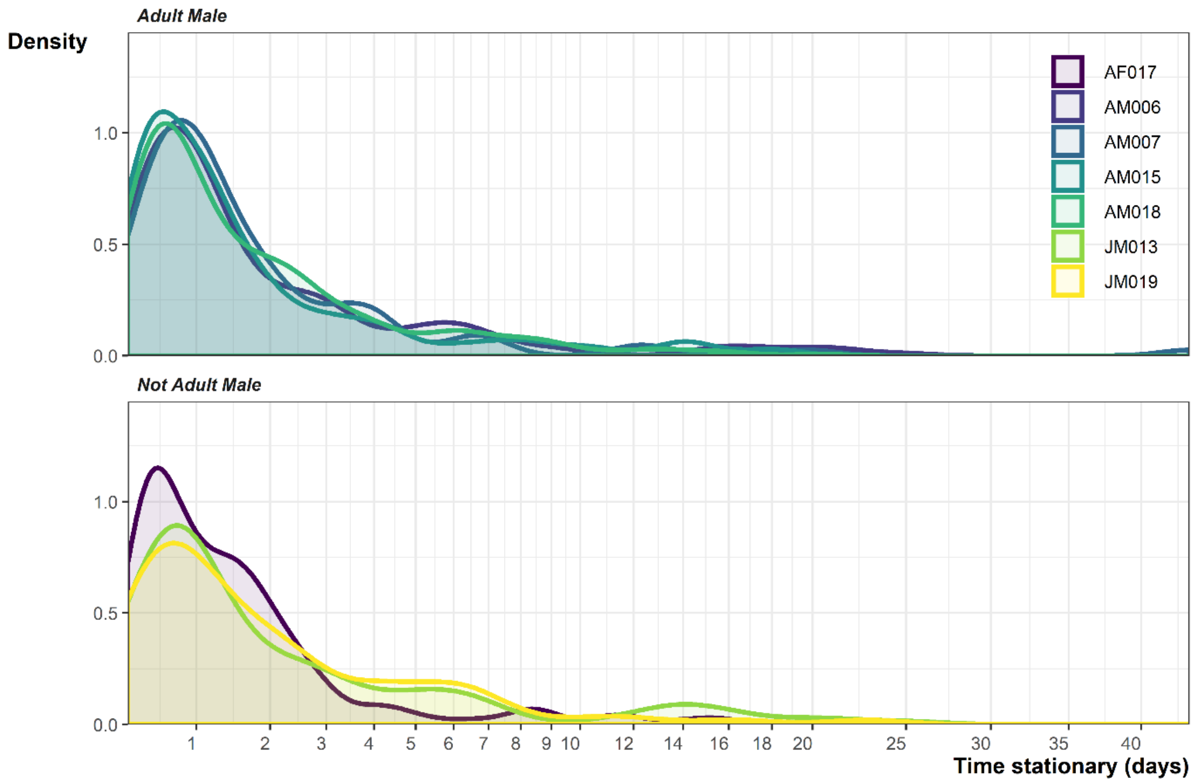

Supplementary Figure 4. Distribution of sheltering periods. To help distinguish individual lines the plots has been split in two. The top plot shows the results from the adult males: AM006, AM007, AM015 and AM018. The lower plot shows AF017, JM013 and JM019.

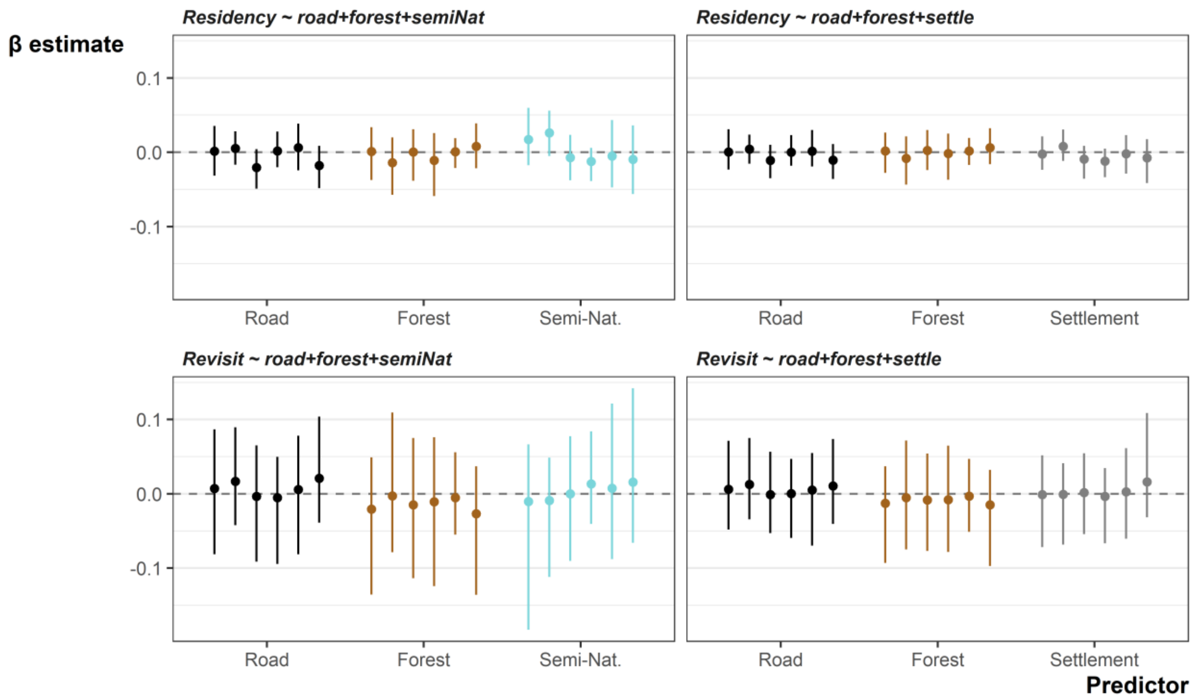

Supplementary Figure 5. Coefficient point estimates and 95% credible intervals from Bayesian regression models. Each point and line denote an individual's point estimate and credible intervals for the impact of distance to landscape feature on residency time and revisit number.

Supplementary Table 1. All coefficient results from Bayesian regression models.

ID	Model	Parameter	Point Estimate	Lower CrI	Upper CrI
AF017	residency_road+forest+semiNat	alpha	1.199	1.165	1.235
AM006	residency_road+forest+semiNat	alpha	1.204	1.170	1.232
AM015	residency_road+forest+semiNat	alpha	1.203	1.172	1.232
AM018	residency_road+forest+semiNat	alpha	1.213	1.180	1.248
JM013	residency_road+forest+semiNat	alpha	1.226	1.187	1.276
JM019	residency_road+forest+semiNat	alpha	1.221	1.188	1.282
AF017	residency_road+forest+semiNat	beta1	0.001	-0.032	0.035
AM006	residency_road+forest+semiNat	beta1	0.005	-0.017	0.028
AM015	residency_road+forest+semiNat	beta1	-0.021	-0.049	0.004
AM018	residency_road+forest+semiNat	beta1	0.002	-0.020	0.028
JM013	residency_road+forest+semiNat	beta1	0.006	-0.024	0.038
JM019	residency_road+forest+semiNat	beta1	-0.018	-0.048	0.009
AF017	residency_road+forest+semiNat	beta2	0.001	-0.037	0.034
AM006	residency_road+forest+semiNat	beta2	-0.014	-0.057	0.020
AM015	residency_road+forest+semiNat	beta2	0.000	-0.038	0.031
AM018	residency_road+forest+semiNat	beta2	-0.011	-0.059	0.026
JM013	residency_road+forest+semiNat	beta2	0.001	-0.021	0.019
JM019	residency_road+forest+semiNat	beta2	0.008	-0.021	0.039
AF017	residency_road+forest+semiNat	beta3	0.017	-0.017	0.060
AM006	residency_road+forest+semiNat	beta3	0.026	-0.005	0.056
AM015	residency_road+forest+semiNat	beta3	-0.007	-0.038	0.023
AM018	residency_road+forest+semiNat	beta3	-0.012	-0.039	0.006
JM013	residency_road+forest+semiNat	beta3	-0.005	-0.047	0.043
JM019	residency_road+forest+semiNat	beta3	-0.010	-0.056	0.036
AF017	residency_road+forest+settle	alpha	1.186	1.159	1.210
AM006	residency_road+forest+settle	alpha	1.205	1.174	1.240
AM015	residency_road+forest+settle	alpha	1.200	1.173	1.226
AM018	residency_road+forest+settle	alpha	1.206	1.179	1.231
JM013	residency_road+forest+settle	alpha	1.228	1.189	1.278
JM019	residency_road+forest+settle	alpha	1.224	1.185	1.266
AF017	residency_road+forest+settle	beta1	0.001	-0.023	0.031
AM006	residency_road+forest+settle	beta1	0.004	-0.016	0.024
AM015	residency_road+forest+settle	beta1	-0.011	-0.035	0.010
AM018	residency_road+forest+settle	beta1	0.000	-0.018	0.023
JM013	residency_road+forest+settle	beta1	0.001	-0.019	0.030
JM019	residency_road+forest+settle	beta1	-0.011	-0.036	0.011
AF017	residency_road+forest+settle	beta2	0.002	-0.028	0.027
AM006	residency_road+forest+settle	beta2	-0.008	-0.044	0.021
AM015	residency_road+forest+settle	beta2	0.003	-0.024	0.030
AM018	residency_road+forest+settle	beta2	-0.002	-0.037	0.025
JM013	residency_road+forest+settle	beta2	0.002	-0.017	0.019
JM019	residency_road+forest+settle	beta2	0.006	-0.016	0.032
AF017	residency_road+forest+settle	beta3	-0.002	-0.024	0.021
AM006	residency_road+forest+settle	beta3	0.008	-0.012	0.031
AM015	residency_road+forest+settle	beta3	-0.009	-0.036	0.008

AM018	residency_road+forest+settle	beta3	-0.012	-0.034	0.005
JM013	residency_road+forest+settle	beta3	-0.002	-0.029	0.023
JM019	residency_road+forest+settle	beta3	-0.007	-0.042	0.017
AF017	revisit_road+forest+semiNat	alpha	0.441	0.258	0.544
AM006	revisit_road+forest+semiNat	alpha	0.143	0.011	0.260
AM015	revisit_road+forest+semiNat	alpha	0.145	0.026	0.253
AM018	revisit_road+forest+semiNat	alpha	0.333	0.206	0.454
JM013	revisit_road+forest+semiNat	alpha	0.167	0.005	0.316
JM019	revisit_road+forest+semiNat	alpha	0.317	0.160	0.486
AF017	revisit_road+forest+semiNat	beta1	0.007	-0.081	0.087
AM006	revisit_road+forest+semiNat	beta1	0.017	-0.042	0.090
AM006	revisit_road+forest+semiNat	beta1	0.017	0.103	0.103
AM015	revisit_road+forest+semiNat	beta1	-0.003	-0.091	0.065
AM018	revisit_road+forest+semiNat	beta1	-0.005	-0.094	0.050
JM013	revisit_road+forest+semiNat	beta1	0.006	-0.081	0.078
JM019	revisit_road+forest+semiNat	beta1	0.021	-0.039	0.104
AF017	revisit_road+forest+semiNat	beta2	-0.021	-0.160	-0.152
AF017	revisit_road+forest+semiNat	beta2	-0.021	-0.136	0.049
AM006	revisit_road+forest+semiNat	beta2	-0.003	-0.079	0.109
AM015	revisit_road+forest+semiNat	beta2	-0.015	-0.114	0.075
AM018	revisit_road+forest+semiNat	beta2	-0.011	-0.124	0.076
JM013	revisit_road+forest+semiNat	beta2	-0.005	-0.055	0.056
JM019	revisit_road+forest+semiNat	beta2	-0.027	-0.136	0.037
AF017	revisit_road+forest+semiNat	beta3	-0.010	-0.183	0.067
AM006	revisit_road+forest+semiNat	beta3	-0.009	-0.112	0.049
AM015	revisit_road+forest+semiNat	beta3	0.000	-0.090	0.077
AM018	revisit_road+forest+semiNat	beta3	0.013	-0.041	0.084
JM013	revisit_road+forest+semiNat	beta3	0.008	-0.088	0.121
JM019	revisit_road+forest+semiNat	beta3	0.016	-0.066	0.142
JM019	revisit_road+forest+semiNat	beta3	0.016	0.145	0.157
AF017	revisit_road+forest+settle	alpha	0.452	0.373	0.529
AM006	revisit_road+forest+settle	alpha	0.138	0.013	0.251
AM015	revisit_road+forest+settle	alpha	0.143	0.020	0.249
AM018	revisit_road+forest+settle	alpha	0.358	0.273	0.443
JM013	revisit_road+forest+settle	alpha	0.161	0.006	0.317
JM019	revisit_road+forest+settle	alpha	0.318	0.170	0.469
AF017	revisit_road+forest+settle	beta1	0.006	-0.062	-0.055
AF017	revisit_road+forest+settle	beta1	0.006	-0.048	0.071
AM006	revisit_road+forest+settle	beta1	0.013	-0.034	0.075
AM015	revisit_road+forest+settle	beta1	-0.001	-0.053	0.057
AM018	revisit_road+forest+settle	beta1	0.000	-0.059	0.047
JM013	revisit_road+forest+settle	beta1	0.005	-0.070	0.055
JM019	revisit_road+forest+settle	beta1	0.011	-0.041	0.074
AF017	revisit_road+forest+settle	beta2	-0.013	-0.093	0.037
AM006	revisit_road+forest+settle	beta2	-0.005	-0.075	0.072
AM015	revisit_road+forest+settle	beta2	-0.008	-0.094	-0.084
AM015	revisit_road+forest+settle	beta2	-0.008	-0.077	0.054
AM018	revisit_road+forest+settle	beta2	-0.008	-0.078	0.065

JM013	revisit_road+forest+settle	beta2	-0.003	-0.051	0.047
JM013	revisit_road+forest+settle	beta2	-0.003	0.050	0.054
JM019	revisit_road+forest+settle	beta2	-0.015	-0.097	0.032
AF017	revisit_road+forest+settle	beta3	-0.001	-0.091	-0.091
AF017	revisit_road+forest+settle	beta3	-0.001	-0.072	0.052
AM006	revisit_road+forest+settle	beta3	-0.001	-0.068	0.041
AM015	revisit_road+forest+settle	beta3	0.002	-0.054	0.055
AM018	revisit_road+forest+settle	beta3	-0.003	-0.067	0.035
JM013	revisit_road+forest+settle	beta3	0.003	-0.060	0.061
JM019	revisit_road+forest+settle	beta3	0.016	-0.032	0.109

Supplementary Table 2. Full ISSF results for all models and individuals.

Term	Estimate	SE	Statistic	p-value	Conf.low	Conf.high	ID	Model	AIC
log_sl	0.002	0.029	0.075	0.940	-0.056	0.060	AM006	model1	5794.50
cos_ta	-0.344	0.215	-1.600	0.110	-0.765	0.077	AM006	model1	5794.50
log_sl:cos_ta	0.068	0.042	1.642	0.101	-0.013	0.150	AM006	model1	5794.50
dist_forest	-2.598	2.784	-0.933	0.351	-8.054	2.858	AM006	model2	5794.62
log_sl	-6.299	3.468	-1.816	0.069	-13.096	0.499	AM006	model2	5794.62
cos_ta	-2.978	8.626	-0.345	0.730	-19.885	13.929	AM006	model2	5794.62
dist_forest:log_sl	0.801	0.441	1.817	0.069	-0.063	1.665	AM006	model2	5794.62
dist_forest:cos_ta	0.332	1.097	0.303	0.762	-1.817	2.481	AM006	model2	5794.62
log_sl:cos_ta	0.074	0.042	1.767	0.077	-0.008	0.156	AM006	model2	5794.62
dist_settle	6.982	3.961	1.763	0.078	-0.782	14.746	AM006	model3	5794.64
log_sl	10.479	5.290	1.981	0.048	0.110	20.848	AM006	model3	5794.64
cos_ta	13.572	10.547	1.287	0.198	-7.099	34.243	AM006	model3	5794.64
dist_settle:log_sl	-1.189	0.600	-1.980	0.048	-2.366	-0.012	AM006	model3	5794.64
dist_settle:cos_ta	-1.578	1.196	-1.320	0.187	-3.921	0.765	AM006	model3	5794.64
log_sl:cos_ta	0.065	0.042	1.571	0.116	-0.016	0.147	AM006	model3	5794.64
dist_semiNat	5.887	1.654	3.558	0.000	2.645	9.130	AM006	model4	5783.74
log_sl	6.911	1.800	3.839	0.000	3.382	10.439	AM006	model4	5783.74
cos_ta	2.157	3.545	0.608	0.543	-4.791	9.104	AM006	model4	5783.74
dist_semiNat:log_sl	-0.787	0.205	-3.843	0.000	-1.188	-0.386	AM006	model4	5783.74
dist_semiNat:cos_ta	-0.280	0.400	-0.699	0.485	-1.065	0.505	AM006	model4	5783.74
log_sl:cos_ta	0.055	0.042	1.290	0.197	-0.028	0.138	AM006	model4	5783.74
dist_road	3.627	1.767	2.053	0.040	0.164	7.090	AM006	model5	5791.24
log_sl	2.716	2.212	1.228	0.220	-1.619	7.051	AM006	model5	5791.24
cos_ta	2.746	4.250	0.646	0.518	-5.584	11.076	AM006	model5	5791.24
dist_road:log_sl	-0.349	0.285	-1.225	0.221	-0.906	0.209	AM006	model5	5791.24
dist_road:cos_ta	-0.396	0.545	-0.727	0.467	-1.463	0.672	AM006	model5	5791.24
log_sl:cos_ta	0.065	0.042	1.548	0.122	-0.017	0.147	AM006	model5	5791.24
dist_water	3.242	1.332	2.434	0.015	0.632	5.853	AM006	model6	5779.88
log_sl	5.799	1.395	4.157	0.000	3.065	8.534	AM006	model6	5779.88
cos_ta	2.130	2.736	0.778	0.436	-3.233	7.492	AM006	model6	5779.88
dist_water:log_sl	-0.677	0.163	-4.163	0.000	-0.996	-0.358	AM006	model6	5779.88
dist_water:cos_ta	-0.291	0.316	-0.921	0.357	-0.909	0.328	AM006	model6	5779.88
log_sl:cos_ta	0.074	0.043	1.746	0.081	-0.009	0.158	AM006	model6	5779.88
dist_road	1.642	0.607	2.704	0.007	0.452	2.833	AM006	model7	5789.37
dist_forest	2.311	1.339	1.725	0.085	-0.315	4.936	AM006	model7	5789.37
dist_semiNat	1.080	1.087	0.994	0.320	-1.049	3.210	AM006	model7	5789.37
log_sl	0.012	0.030	0.402	0.687	-0.046	0.071	AM006	model7	5789.37
cos_ta	-0.336	0.219	-1.529	0.126	-0.766	0.095	AM006	model7	5789.37
log_sl:cos_ta	0.065	0.043	1.496	0.135	-0.020	0.150	AM006	model7	5789.37
dist_road	1.719	0.615	2.796	0.005	0.514	2.924	AM006	model8	5789.90
dist_forest	2.172	1.329	1.634	0.102	-0.434	4.777	AM006	model8	5789.90
dist_settle	-1.019	1.487	-0.685	0.493	-3.932	1.895	AM006	model8	5789.90
log_sl	0.012	0.030	0.386	0.699	-0.047	0.070	AM006	model8	5789.90
cos_ta	-0.372	0.217	-1.711	0.087	-0.798	0.054	AM006	model8	5789.90
log_sl:cos_ta	0.076	0.042	1.806	0.071	-0.007	0.159	AM006	model8	5789.90

dist_road	1.593	0.609	2.617	0.009	0.400	2.786	AM006	model9	5789.75
dist_forest	2.091	1.335	1.566	0.117	-0.526	4.708	AM006	model9	5789.75
dist_water	-0.693	0.877	-0.790	0.430	-2.412	1.026	AM006	model9	5789.75
log_sl	0.012	0.030	0.387	0.699	-0.047	0.070	AM006	model9	5789.75
cos_ta	-0.391	0.220	-1.779	0.075	-0.821	0.040	AM006	model9	5789.75
log_sl:cos_ta	0.082	0.043	1.898	0.058	-0.003	0.168	AM006	model9	5789.75
log_sl	0.002	0.026	0.096	0.923	-0.048	0.053	AM015	model1	6205.87
cos_ta	-0.390	0.180	-2.164	0.030	-0.743	-0.037	AM015	model1	6205.87
log_sl:cos_ta	0.081	0.037	2.229	0.026	0.010	0.153	AM015	model1	6205.87
dist_forest	-2.268	2.040	-1.112	0.266	-6.265	1.730	AM015	model2	6187.03
log_sl	-8.003	2.498	-3.204	0.001	-12.899	-3.107	AM015	model2	6187.03
cos_ta	5.667	6.159	0.920	0.358	-6.404	17.738	AM015	model2	6187.03
dist_forest:log_sl	1.023	0.319	3.205	0.001	0.397	1.648	AM015	model2	6187.03
dist_forest:cos_ta	-0.784	0.788	-0.995	0.320	-2.328	0.761	AM015	model2	6187.03
log_sl:cos_ta	0.110	0.037	2.956	0.003	0.037	0.182	AM015	model2	6187.03
dist_settle	12.938	4.199	3.081	0.002	4.708	21.169	AM015	model3	6201.81
log_sl	16.907	5.603	3.018	0.003	5.926	27.888	AM015	model3	6201.81
cos_ta	-1.962	11.454	-0.171	0.864	-24.412	20.488	AM015	model3	6201.81
dist_settle:log_sl	-1.914	0.634	-3.018	0.003	-3.156	-0.671	AM015	model3	6201.81
dist_settle:cos_ta	0.178	1.295	0.138	0.890	-2.359	2.716	AM015	model3	6201.81
log_sl:cos_ta	0.080	0.036	2.181	0.029	0.008	0.151	AM015	model3	6201.81
dist_semiNat	9.867	2.043	4.829	0.000	5.863	13.872	AM015	model4	6186.73
log_sl	4.790	2.413	1.985	0.047	0.060	9.519	AM015	model4	6186.73
cos_ta	-6.860	5.069	-1.353	0.176	-16.795	3.076	AM015	model4	6186.73
dist_semiNat:log_sl	-0.537	0.271	-1.981	0.048	-1.069	-0.006	AM015	model4	6186.73
dist_semiNat:cos_ta	0.733	0.568	1.289	0.197	-0.381	1.846	AM015	model4	6186.73
log_sl:cos_ta	0.063	0.037	1.677	0.094	-0.011	0.136	AM015	model4	6186.73
dist_road	4.468	1.821	2.454	0.014	0.899	8.036	AM015	model5	6201.98
log_sl	6.556	2.211	2.965	0.003	2.223	10.890	AM015	model5	6201.98
cos_ta	0.520	4.453	0.117	0.907	-8.208	9.247	AM015	model5	6201.98
dist_road:log_sl	-0.844	0.285	-2.967	0.003	-1.402	-0.286	AM015	model5	6201.98
dist_road:cos_ta	-0.117	0.571	-0.205	0.838	-1.236	1.003	AM015	model5	6201.98
log_sl:cos_ta	0.081	0.036	2.233	0.026	0.010	0.153	AM015	model5	6201.98
dist_water	5.191	1.509	3.440	0.001	2.233	8.148	AM015	model6	6199.01
log_sl	4.162	1.602	2.599	0.009	1.023	7.301	AM015	model6	6199.01
cos_ta	-4.428	3.341	-1.325	0.185	-10.976	2.120	AM015	model6	6199.01
dist_water:log_sl	-0.479	0.184	-2.599	0.009	-0.840	-0.118	AM015	model6	6199.01
dist_water:cos_ta	0.467	0.383	1.221	0.222	-0.283	1.217	AM015	model6	6199.01
log_sl:cos_ta	0.073	0.037	1.960	0.050	0.000	0.145	AM015	model6	6199.01
dist_road	0.763	0.729	1.047	0.295	-0.666	2.192	AM015	model7	6171.16
dist_forest	4.143	0.962	4.306	0.000	2.257	6.028	AM015	model7	6171.16
dist_semiNat	8.340	1.634	5.104	0.000	5.138	11.542	AM015	model7	6171.16
log_sl	0.023	0.027	0.884	0.377	-0.029	0.075	AM015	model7	6171.16
cos_ta	-0.388	0.185	-2.095	0.036	-0.751	-0.025	AM015	model7	6171.16
log_sl:cos_ta	0.080	0.038	2.091	0.037	0.005	0.155	AM015	model7	6171.16
dist_road	-0.642	0.776	-0.827	0.408	-2.163	0.879	AM015	model8	6195.03
dist_forest	3.296	0.911	3.618	0.000	1.511	5.082	AM015	model8	6195.03
dist_settle	2.600	1.993	1.305	0.192	-1.306	6.507	AM015	model8	6195.03

log_sl	0.011	0.026	0.438	0.662	-0.040	0.063	AM015	model8	6195.03
cos_ta	-0.450	0.182	-2.468	0.014	-0.808	-0.093	AM015	model8	6195.03
log_sl:cos_ta	0.103	0.037	2.758	0.006	0.030	0.177	AM015	model8	6195.03
dist_road	-0.043	0.695	-0.062	0.950	-1.406	1.319	AM015	model9	6187.41
dist_forest	3.947	0.952	4.144	0.000	2.080	5.814	AM015	model9	6187.41
dist_water	3.606	1.178	3.062	0.002	1.298	5.914	AM015	model9	6187.41
log_sl	0.016	0.026	0.589	0.556	-0.036	0.067	AM015	model9	6187.41
cos_ta	-0.411	0.184	-2.236	0.025	-0.772	-0.051	AM015	model9	6187.41
log_sl:cos_ta	0.090	0.038	2.359	0.018	0.015	0.164	AM015	model9	6187.41
log_sl	0.001	0.025	0.038	0.970	-0.048	0.050	AF017	model1	7573.47
cos_ta	-0.347	0.147	-2.358	0.018	-0.635	-0.059	AF017	model1	7573.47
log_sl:cos_ta	0.082	0.035	2.320	0.020	0.013	0.151	AF017	model1	7573.47
dist_forest	6.749	2.642	2.554	0.011	1.570	11.928	AF017	model2	7555.47
log_sl	2.564	3.675	0.698	0.485	-4.638	9.766	AF017	model2	7555.47
cos_ta	-6.687	7.388	-0.905	0.365	-21.168	7.794	AF017	model2	7555.47
dist_forest:log_sl	-0.327	0.471	-0.694	0.488	-1.249	0.596	AF017	model2	7555.47
dist_forest:cos_ta	0.804	0.946	0.850	0.395	-1.049	2.657	AF017	model2	7555.47
log_sl:cos_ta	0.113	0.036	3.134	0.002	0.043	0.184	AF017	model2	7555.47
dist_settle	-11.608	6.486	-1.790	0.073	-24.321	1.104	AF017	model3	7542.05
log_sl	15.698	9.068	1.731	0.083	-2.075	33.471	AF017	model3	7542.05
cos_ta	17.120	17.916	0.956	0.339	-17.994	52.234	AF017	model3	7542.05
dist_settle:log_sl	-1.772	1.025	-1.730	0.084	-3.780	0.236	AF017	model3	7542.05
dist_settle:cos_ta	-1.979	2.024	-0.978	0.328	-5.946	1.988	AF017	model3	7542.05
log_sl:cos_ta	0.110	0.036	3.063	0.002	0.039	0.180	AF017	model3	7542.05
dist_semiNat	49.638	6.039	8.219	0.000	37.801	61.475	AF017	model4	7492.52
log_sl	5.493	9.832	0.559	0.576	-13.778	24.763	AF017	model4	7492.52
cos_ta	-22.353	19.731	-1.133	0.257	-61.025	16.319	AF017	model4	7492.52
dist_semiNat:log_sl	-0.609	1.098	-0.555	0.579	-2.760	1.543	AF017	model4	7492.52
dist_semiNat:cos_ta	2.458	2.202	1.116	0.264	-1.857	6.773	AF017	model4	7492.52
log_sl:cos_ta	0.078	0.036	2.148	0.032	0.007	0.150	AF017	model4	7492.52
dist_road	3.042	3.462	0.879	0.380	-3.744	9.828	AF017	model5	7557.50
log_sl	12.230	4.620	2.647	0.008	3.175	21.284	AF017	model5	7557.50
cos_ta	4.479	8.592	0.521	0.602	-12.362	21.320	AF017	model5	7557.50
dist_road:log_sl	-1.566	0.592	-2.646	0.008	-2.725	-0.406	AF017	model5	7557.50
dist_road:cos_ta	-0.621	1.100	-0.565	0.572	-2.776	1.534	AF017	model5	7557.50
log_sl:cos_ta	0.093	0.035	2.634	0.008	0.024	0.163	AF017	model5	7557.50
dist_water	13.119	4.678	2.804	0.005	3.950	22.288	AF017	model6	7566.34
log_sl	19.036	6.296	3.024	0.002	6.696	31.376	AF017	model6	7566.34
cos_ta	14.500	10.794	1.343	0.179	-6.657	35.657	AF017	model6	7566.34
dist_water:log_sl	-2.172	0.718	-3.024	0.002	-3.580	-0.764	AF017	model6	7566.34
dist_water:cos_ta	-1.692	1.231	-1.375	0.169	-4.104	0.720	AF017	model6	7566.34
log_sl:cos_ta	0.073	0.036	2.049	0.040	0.003	0.143	AF017	model6	7566.34
dist_road	-0.652	1.678	-0.389	0.698	-3.940	2.637	AF017	model7	7471.48
dist_forest	5.103	1.326	3.847	0.000	2.503	7.703	AF017	model7	7471.48
dist_semiNat	46.099	4.885	9.437	0.000	36.525	55.673	AF017	model7	7471.48
log_sl	0.052	0.026	1.948	0.051	0.000	0.103	AF017	model7	7471.48
cos_ta	-0.400	0.153	-2.612	0.009	-0.699	-0.100	AF017	model7	7471.48
log_sl:cos_ta	0.110	0.037	2.929	0.003	0.036	0.183	AF017	model7	7471.48

dist_road	-1.496	1.627	-0.920	0.358	-4.684	1.693	AF017	model8	7539.43
dist_forest	2.511	1.294	1.940	0.052	-0.025	5.048	AF017	model8	7539.43
dist_settle	-16.100	4.265	-3.775	0.000	-24.459	-7.741	AF017	model8	7539.43
log_sl	0.019	0.025	0.750	0.453	-0.031	0.069	AF017	model8	7539.43
cos_ta	-0.430	0.149	-2.880	0.004	-0.722	-0.137	AF017	model8	7539.43
log_sl:cos_ta	0.125	0.036	3.443	0.001	0.054	0.197	AF017	model8	7539.43
dist_road	-2.641	1.635	-1.616	0.106	-5.846	0.563	AF017	model9	7530.33
dist_forest	8.751	1.593	5.495	0.000	5.630	11.872	AF017	model9	7530.33
dist_water	16.967	3.491	4.860	0.000	10.124	23.810	AF017	model9	7530.33
log_sl	0.027	0.026	1.059	0.290	-0.023	0.078	AF017	model9	7530.33
cos_ta	-0.398	0.151	-2.641	0.008	-0.693	-0.103	AF017	model9	7530.33
log_sl:cos_ta	0.105	0.037	2.858	0.004	0.033	0.178	AF017	model9	7530.33
log_sl	-0.006	0.040	-0.146	0.884	-0.085	0.073	JM013	model1	4014.21
cos_ta	-0.289	0.277	-1.043	0.297	-0.831	0.254	JM013	model1	4014.21
log_sl:cos_ta	0.057	0.057	0.993	0.321	-0.055	0.169	JM013	model1	4014.21
dist_forest	0.109	1.122	0.097	0.923	-2.091	2.308	JM013	model2	4013.58
log_sl	-0.721	1.243	-0.580	0.562	-3.157	1.715	JM013	model2	4013.58
cos_ta	-4.996	2.311	-2.162	0.031	-9.525	-0.467	JM013	model2	4013.58
dist_forest:log_sl	0.095	0.163	0.580	0.562	-0.225	0.414	JM013	model2	4013.58
dist_forest:cos_ta	0.614	0.302	2.031	0.042	0.022	1.206	JM013	model2	4013.58
log_sl:cos_ta	0.067	0.059	1.141	0.254	-0.048	0.181	JM013	model2	4013.58
dist_settle	3.421	5.699	0.600	0.548	-7.749	14.591	JM013	model3	4014.60
log_sl	4.489	8.144	0.551	0.581	-11.472	20.451	JM013	model3	4014.60
cos_ta	32.701	14.761	2.215	0.027	3.769	61.633	JM013	model3	4014.60
dist_settle:log_sl	-0.508	0.921	-0.552	0.581	-2.313	1.297	JM013	model3	4014.60
dist_settle:cos_ta	-3.730	1.669	-2.235	0.025	-7.000	-0.460	JM013	model3	4014.60
log_sl:cos_ta	0.052	0.058	0.907	0.364	-0.061	0.165	JM013	model3	4014.60
dist_semiNat	-0.565	4.585	-0.123	0.902	-9.552	8.423	JM013	model4	4017.76
log_sl	-1.083	6.436	-0.168	0.866	-13.697	11.532	JM013	model4	4017.76
cos_ta	18.186	11.936	1.524	0.128	-5.208	41.580	JM013	model4	4017.76
dist_semiNat:log_sl	0.122	0.725	0.168	0.867	-1.299	1.542	JM013	model4	4017.76
dist_semiNat:cos_ta	-2.080	1.344	-1.548	0.122	-4.713	0.553	JM013	model4	4017.76
log_sl:cos_ta	0.056	0.058	0.976	0.329	-0.057	0.170	JM013	model4	4017.76
dist_road	-0.205	3.231	-0.064	0.949	-6.537	6.127	JM013	model5	4017.31
log_sl	0.660	4.421	0.149	0.881	-8.004	9.324	JM013	model5	4017.31
cos_ta	11.256	7.639	1.473	0.141	-3.717	26.229	JM013	model5	4017.31
dist_road:log_sl	-0.085	0.568	-0.150	0.881	-1.199	1.029	JM013	model5	4017.31
dist_road:cos_ta	-1.485	0.982	-1.513	0.130	-3.409	0.439	JM013	model5	4017.31
log_sl:cos_ta	0.058	0.057	1.007	0.314	-0.055	0.170	JM013	model5	4017.31
dist_water	15.882	6.458	2.459	0.014	3.225	28.539	JM013	model6	4013.23
log_sl	19.349	9.910	1.953	0.051	-0.073	38.772	JM013	model6	4013.23
cos_ta	-12.201	17.538	-0.696	0.487	-46.574	22.173	JM013	model6	4013.23
dist_water:log_sl	-2.204	1.128	-1.953	0.051	-4.415	0.007	JM013	model6	4013.23
dist_water:cos_ta	1.355	1.994	0.680	0.497	-2.553	5.263	JM013	model6	4013.23
log_sl:cos_ta	0.059	0.057	1.030	0.303	-0.053	0.171	JM013	model6	4013.23
dist_road	-0.647	1.040	-0.622	0.534	-2.685	1.391	JM013	model7	4017.55
dist_forest	1.011	0.708	1.427	0.154	-0.378	2.399	JM013	model7	4017.55
dist_semiNat	1.877	2.773	0.677	0.498	-3.558	7.312	JM013	model7	4017.55

log_sl	-0.004	0.040	-0.100	0.921	-0.083	0.075	JM013	model7	4017.55
cos_ta	-0.330	0.279	-1.182	0.237	-0.877	0.217	JM013	model7	4017.55
log_sl:cos_ta	0.070	0.058	1.202	0.229	-0.044	0.185	JM013	model7	4017.55
dist_road	-0.509	1.034	-0.492	0.622	-2.535	1.517	JM013	model8	4017.61
dist_forest	0.983	0.697	1.410	0.159	-0.384	2.350	JM013	model8	4017.61
dist_settle	1.914	3.028	0.632	0.527	-4.021	7.849	JM013	model8	4017.61
log_sl	-0.005	0.040	-0.113	0.910	-0.083	0.074	JM013	model8	4017.61
cos_ta	-0.334	0.279	-1.200	0.230	-0.881	0.212	JM013	model8	4017.61
log_sl:cos_ta	0.072	0.058	1.234	0.217	-0.042	0.186	JM013	model8	4017.61
dist_road	-0.624	1.034	-0.604	0.546	-2.652	1.403	JM013	model9	4015.71
dist_forest	0.704	0.646	1.090	0.276	-0.562	1.971	JM013	model9	4015.71
dist_water	4.254	2.806	1.516	0.130	-1.247	9.755	JM013	model9	4015.71
log_sl	-0.003	0.040	-0.079	0.937	-0.082	0.076	JM013	model9	4015.71
cos_ta	-0.326	0.279	-1.168	0.243	-0.873	0.221	JM013	model9	4015.71
log_sl:cos_ta	0.069	0.058	1.181	0.238	-0.045	0.183	JM013	model9	4015.71
log_sl	-0.001	0.041	-0.036	0.971	-0.082	0.079	JM019	model1	2402.41
cos_ta	-0.209	0.269	-0.776	0.437	-0.737	0.319	JM019	model1	2402.41
log_sl:cos_ta	0.048	0.058	0.826	0.409	-0.066	0.163	JM019	model1	2402.41
dist_forest	1.300	1.837	0.708	0.479	-2.299	4.900	JM019	model2	2399.25
log_sl	-3.101	2.029	-1.528	0.127	-7.079	0.877	JM019	model2	2399.25
cos_ta	-5.265	5.065	-1.039	0.299	-15.192	4.662	JM019	model2	2399.25
dist_forest:log_sl	0.398	0.261	1.529	0.126	-0.112	0.909	JM019	model2	2399.25
dist_forest:cos_ta	0.649	0.650	0.998	0.318	-0.625	1.922	JM019	model2	2399.25
log_sl:cos_ta	0.046	0.059	0.773	0.440	-0.070	0.162	JM019	model2	2399.25
dist_settle	11.810	5.934	1.990	0.047	0.179	23.441	JM019	model3	2402.45
log_sl	14.968	7.738	1.934	0.053	-0.198	30.134	JM019	model3	2402.45
cos_ta	19.452	16.343	1.190	0.234	-12.581	51.484	JM019	model3	2402.45
dist_settle:log_sl	-1.695	0.876	-1.935	0.053	-3.413	0.022	JM019	model3	2402.45
dist_settle:cos_ta	-2.223	1.848	-1.203	0.229	-5.846	1.399	JM019	model3	2402.45
log_sl:cos_ta	0.034	0.059	0.579	0.562	-0.082	0.150	JM019	model3	2402.45
dist_semiNat	17.157	4.575	3.750	0.000	8.190	26.123	JM019	model4	2392.32
log_sl	10.508	6.093	1.725	0.085	-1.434	22.451	JM019	model4	2392.32
cos_ta	10.952	12.849	0.852	0.394	-14.231	36.135	JM019	model4	2392.32
dist_semiNat:log_sl	-1.179	0.684	-1.724	0.085	-2.519	0.162	JM019	model4	2392.32
dist_semiNat:cos_ta	-1.251	1.439	-0.869	0.385	-4.071	1.569	JM019	model4	2392.32
log_sl:cos_ta	0.042	0.059	0.708	0.479	-0.074	0.158	JM019	model4	2392.32
dist_road	1.191	1.989	0.599	0.549	-2.708	5.090	JM019	model5	2406.31
log_sl	2.312	2.248	1.028	0.304	-2.095	6.719	JM019	model5	2406.31
cos_ta	3.119	4.759	0.656	0.512	-6.208	12.446	JM019	model5	2406.31
dist_road:log_sl	-0.300	0.292	-1.029	0.303	-0.872	0.272	JM019	model5	2406.31
dist_road:cos_ta	-0.433	0.615	-0.704	0.481	-1.638	0.772	JM019	model5	2406.31
log_sl:cos_ta	0.051	0.059	0.862	0.389	-0.065	0.166	JM019	model5	2406.31
dist_water	6.567	3.990	1.646	0.100	-1.252	14.387	JM019	model6	2404.11
log_sl	6.300	4.933	1.277	0.202	-3.368	15.968	JM019	model6	2404.11
cos_ta	13.353	11.228	1.189	0.234	-8.655	35.360	JM019	model6	2404.11
dist_water:log_sl	-0.721	0.565	-1.277	0.201	-1.828	0.385	JM019	model6	2404.11
dist_water:cos_ta	-1.550	1.284	-1.208	0.227	-4.066	0.966	JM019	model6	2404.11
log_sl:cos_ta	0.038	0.059	0.651	0.515	-0.077	0.153	JM019	model6	2404.11

dist_road	-1.449	1.080	-1.342	0.180	-3.567	0.668	JM019	model7	2388.28
dist_forest	3.354	1.381	2.428	0.015	0.647	6.061	JM019	model7	2388.28
dist_semiNat	12.804	3.405	3.760	0.000	6.130	19.477	JM019	model7	2388.28
log_sl	0.020	0.042	0.472	0.637	-0.063	0.102	JM019	model7	2388.28
cos_ta	-0.252	0.275	-0.917	0.359	-0.791	0.287	JM019	model7	2388.28
log_sl:cos_ta	0.065	0.061	1.078	0.281	-0.053	0.184	JM019	model7	2388.28
dist_road	-2.259	1.465	-1.543	0.123	-5.130	0.611	JM019	model8	2400.04
dist_forest	3.162	1.349	2.344	0.019	0.518	5.807	JM019	model8	2400.04
dist_settle	7.222	4.797	1.506	0.132	-2.180	16.625	JM019	model8	2400.04
log_sl	0.009	0.042	0.209	0.834	-0.073	0.090	JM019	model8	2400.04
cos_ta	-0.210	0.272	-0.774	0.439	-0.744	0.323	JM019	model8	2400.04
log_sl:cos_ta	0.048	0.060	0.801	0.423	-0.069	0.165	JM019	model8	2400.04
dist_road	-1.771	1.075	-1.648	0.099	-3.877	0.335	JM019	model9	2398.12
dist_forest	3.758	1.402	2.680	0.007	1.010	6.506	JM019	model9	2398.12
dist_water	6.688	3.187	2.098	0.036	0.441	12.934	JM019	model9	2398.12
log_sl	0.009	0.042	0.208	0.835	-0.073	0.090	JM019	model9	2398.12
cos_ta	-0.220	0.271	-0.811	0.417	-0.751	0.311	JM019	model9	2398.12
log_sl:cos_ta	0.053	0.059	0.890	0.374	-0.064	0.169	JM019	model9	2398.12
log_sl	-0.001	0.017	-0.049	0.961	-0.035	0.033	AM018	model1	10567.77
cos_ta	-0.179	0.118	-1.517	0.129	-0.410	0.052	AM018	model1	10567.77
log_sl:cos_ta	0.037	0.024	1.530	0.126	-0.010	0.085	AM018	model1	10567.77
dist_forest	0.203	4.978	0.041	0.968	-9.554	9.959	AM018	model2	10530.19
log_sl	-19.517	6.817	-2.863	0.004	-32.878	-6.156	AM018	model2	10530.19
cos_ta	30.593	22.751	1.345	0.179	-13.998	75.184	AM018	model2	10530.19
dist_forest:log_sl	2.475	0.864	2.864	0.004	0.781	4.169	AM018	model2	10530.19
dist_forest:cos_ta	-3.905	2.884	-1.354	0.176	-9.557	1.748	AM018	model2	10530.19
log_sl:cos_ta	0.050	0.025	2.028	0.043	0.002	0.099	AM018	model2	10530.19
dist_settle	3.389	2.610	1.299	0.194	-1.726	8.505	AM018	model3	10552.30
log_sl	-3.141	3.823	-0.822	0.411	-10.634	4.352	AM018	model3	10552.30
cos_ta	-18.552	10.014	-1.853	0.064	-38.180	1.076	AM018	model3	10552.30
dist_settle:log_sl	0.356	0.433	0.823	0.411	-0.492	1.205	AM018	model3	10552.30
dist_settle:cos_ta	2.081	1.134	1.835	0.066	-0.141	4.303	AM018	model3	10552.30
log_sl:cos_ta	0.035	0.025	1.437	0.151	-0.013	0.083	AM018	model3	10552.30
dist_semiNat	0.915	1.117	0.819	0.413	-1.275	3.105	AM018	model4	10563.71
log_sl	2.765	1.095	2.526	0.012	0.619	4.910	AM018	model4	10563.71
cos_ta	2.237	2.743	0.815	0.415	-3.139	7.612	AM018	model4	10563.71
dist_semiNat:log_sl	-0.319	0.126	-2.528	0.011	-0.566	-0.072	AM018	model4	10563.71
dist_semiNat:cos_ta	-0.280	0.315	-0.887	0.375	-0.898	0.338	AM018	model4	10563.71
log_sl:cos_ta	0.042	0.025	1.690	0.091	-0.007	0.090	AM018	model4	10563.71
dist_road	5.911	1.508	3.920	0.000	2.955	8.867	AM018	model5	10498.78
log_sl	1.082	2.065	0.524	0.600	-2.966	5.130	AM018	model5	10498.78
cos_ta	-6.882	4.964	-1.386	0.166	-16.611	2.847	AM018	model5	10498.78
dist_road:log_sl	-0.135	0.264	-0.512	0.608	-0.653	0.382	AM018	model5	10498.78
dist_road:cos_ta	0.855	0.633	1.351	0.177	-0.386	2.096	AM018	model5	10498.78
log_sl:cos_ta	0.042	0.025	1.673	0.094	-0.007	0.092	AM018	model5	10498.78
dist_water	2.708	1.305	2.075	0.038	0.150	5.267	AM018	model6	10559.55
log_sl	4.721	1.437	3.286	0.001	1.905	7.537	AM018	model6	10559.55
cos_ta	2.857	3.416	0.836	0.403	-3.838	9.552	AM018	model6	10559.55

dist_water:log_sl	-0.551	0.168	-3.289	0.001	-0.879	-0.223	AM018	model6	10559.55
dist_water:cos_ta	-0.355	0.397	-0.893	0.372	-1.134	0.424	AM018	model6	10559.55
log_sl:cos_ta	0.040	0.025	1.616	0.106	-0.008	0.088	AM018	model6	10559.55
dist_road	5.118	0.653	7.834	0.000	3.838	6.399	AM018	model7	10471.26
dist_forest	9.739	2.360	4.127	0.000	5.114	14.365	AM018	model7	10471.26
dist_semiNat	-1.743	0.831	-2.098	0.036	-3.371	-0.115	AM018	model7	10471.26
log_sl	0.034	0.018	1.840	0.066	-0.002	0.069	AM018	model7	10471.26
cos_ta	-0.228	0.123	-1.854	0.064	-0.469	0.013	AM018	model7	10471.26
log_sl:cos_ta	0.057	0.026	2.217	0.027	0.007	0.108	AM018	model7	10471.26
dist_road	4.515	0.677	6.672	0.000	3.189	5.841	AM018	model8	10472.37
dist_forest	10.353	2.358	4.391	0.000	5.732	14.975	AM018	model8	10472.37
dist_settle	2.504	1.402	1.786	0.074	-0.244	5.253	AM018	model8	10472.37
log_sl	0.034	0.018	1.871	0.061	-0.002	0.070	AM018	model8	10472.37
cos_ta	-0.207	0.123	-1.688	0.091	-0.448	0.033	AM018	model8	10472.37
log_sl:cos_ta	0.048	0.026	1.868	0.062	-0.002	0.098	AM018	model8	10472.37
dist_road	4.981	0.647	7.699	0.000	3.713	6.248	AM018	model9	10474.02
dist_forest	9.954	2.369	4.201	0.000	5.310	14.598	AM018	model9	10474.02
dist_water	-0.966	0.759	-1.274	0.203	-2.453	0.521	AM018	model9	10474.02
log_sl	0.033	0.018	1.801	0.072	-0.003	0.068	AM018	model9	10474.02
cos_ta	-0.220	0.123	-1.789	0.074	-0.461	0.021	AM018	model9	10474.02
log_sl:cos_ta	0.053	0.026	2.073	0.038	0.003	0.104	AM018	model9	10474.02
